# Practical Aids to Beauty

**Published:** 1889-11

**Authors:** 


### PRACTICAL AIDS TO BEAUTY.

An offensive breath will spoil the prettiest girl. When it arises from the teeth the dentist alone can permanently remove the cause, but when trouble arises from a disordered stomach, dissolve a teaspoonful of crystal of permanganate of potash in two ounces of water, and put enough of it in water to rinse the mouth to make it pink ; do not swallow the water, but rinse the mouth and cleanse the teeth. This, like the bichloride of mercury lotion, is a poison and should be kept out of the reach of children, and where it will not be mistaken for medicine. When the teeth are discolored, finely pulverised pumice stone is preferable to charcoal powder for removing the discoloration. If the gums are tender a few drops of tincture of myrrh, dropped into the water with which the mouth is rinsed, will be found excellent. The chalk and myrrh dentrifice to be procured at almost any drug store, is delightful and very beneficial, which cannot be said of all dentifrices, many of them contain acid, which in time destroyes the enamel. The teeth should receive attention after each meal, and the last thing before retiring. Wooden toothpicks should be kept conveniently within the reach of the smallest troddler. A thread of white sadlers silk, of coarse twist, is excellent to remove food from between close teeth.

